# Chronic hepatitis B infection and non-hepatocellular cancers: A hospital registry-based, case-control study

**DOI:** 10.1371/journal.pone.0193232

**Published:** 2018-03-15

**Authors:** Jihyun An, Jong Woo Kim, Ju Hyun Shim, Seungbong Han, Chang Sik Yu, Jaewon Choe, Danbi Lee, Kang Mo Kim, Young-Suk Lim, Young-Hwa Chung, Yung Sang Lee, Dong Jin Suh, Jin Hyoung Kim, Han Chu Lee

**Affiliations:** 1 Department of Gastroenterology, Asan Liver Center, Asan Medical Center, University of Ulsan College of Medicine, Seoul, Republic of Korea; 2 Department of Radiology and Research Institute of Radiology, Asan Liver Center, Asan Medical Center, University of Ulsan College of Medicine, Seoul, Republic of Korea; 3 Department of Applied Statistics, Gachon University, Seongnam, Gyeonggi-do, Republic of Korea; 4 Department of Colon and Rectal Surgery, Asan Medical Center, University of Ulsan College of Medicine, Seoul, Republic of Korea; 5 The Health Screening and Promotion Center, Asan Medical Center, University of Ulsan College of Medicine, Seoul, Republic of Korea; 6 Department of Internal Medicine, Vievisnamuh Hospital, Seoul, Republic of Korea; Centers for Disease Control and Prevention, UNITED STATES

## Abstract

**Background:**

Prior epidemiological evidences suggest that hepatitis B virus (HBV) infection is linked to cancers other than hepatocellular carcinoma. This prospective hospital registry-based case-control study aimed to investigate the sero-epidemiological association between chronic HBV infection and various types of cancer.

**Methods:**

95,034 patients with first-diagnosed non-hepatocellular malignancy in a tertiary hospital between 2007 and 2014; and 118,891 non-cancer individuals as controls from a health promotion center were included. Cases and controls were compared for HBV surface antigen (HBsAg) positivity by conditional regression with adjustment for age, hypertension, diabetes, body mass index, alcohol consumption, smoking status and cholesterol level in both genders.

**Results:**

An analysis of matched data indicated significant associations of HBV infection with lymphoma (adjusted odds ratio[AOR] 1.53 [95% CI 1.12–2.09] in men and 3.04 [1.92–4.82] in women) and biliary cancer (2.59[1.98–3.39] in men and 1.71[1.16–2.51] in women). Cervical (1.49[1.11–2.00]), uterine (1.69[1.09–2.61]), breast (1.16[1.02–1.32]), thyroid (1.49[1.28–1.74]), and lung cancers (1.79[1.32–2.44]) in women; and skin cancer (5.33[1.55–18.30]) in men were also significantly related to HBV infection.

**Conclusions:**

Chronic HBV infection is associated with several malignant disorders including lymphoma, and biliary, cervical, uterine, breast, thyroid, lung, and skin cancers. Our findings may offer additional insights into the development of these neoplasms and may suggest the need to consider HBV screening in cancer patients and cancer surveillance in HBV-infected subjects.

## Introduction

Infection with hepatitis B virus (HBV) is a major global public health challenge with a broad spectrum of clinical manifestations, and approximately 240 million individuals are infected with this virus.[1] HBV displays a substantial hepatic tissue tropism, and can induce chronic liver damage and subsequent hepatocarcinogenesis. [2, 3]

In addition to a potential to produce cholangiocarcinomas, prior epidemiological reports have suggested that chronic HBV infection also increases the risk of extra-hepatic malignancies such as non-Hodgkin lymphoma (NHL), pancreatic cancer, and gastric cancer. [4–7] Such associations could be explained by several biological effects of HBV: 1) As observed in autopsies, HBV may move systemically through the bloodstream and infect non-hepatic tissues including lymph nodes, kidney, pancreas, and gastrointestinal tract [8–10]; 2) given the existence of viral DNA and antigens in organ sites other than liver, HBV may replicate within such extra-hepatic reservoirs and play oncogenic roles [11–16]; 3) HBV may target organs adjacent to the liver, such as pancreas and stomach, which share common vascular and bile duct systems [17]; and 4) chronic immune stimulation of B-lymphocytes associated with persistent hepatic infection may be directly involved in lymphomagenesis.[18]

To date, most of studies of the linkage between HBV infection and extra-hepatic cancers have involved population-based cohorts and case-control series with only a limited number of types of malignancy.[4, 5, 7, 19, 20] Moreover, per-cancer observations derived from these studies were not consistent across publications, because of differences between the studied populations in terms of prevalence of HBV, screening practices, and monitoring duration, together with differences of ethnicity and exposure to carcinogens.[21, 22] The information obtained could point to a clinical need for periodic surveillance of HBV carriers for cancers at specific sites or of particular types, as already occurs globally in the case of hepatocellular carcinoma (HCC), and provide insight into the development of various malignancies.

Considering the potential benefit of the relevant findings, we aimed to investigate the sero-epidemiological connections between infection with HBV and various malignancies other than HCC, using data from an ongoing prospective registry of cancer patients, along with control data for a non-cancerous population in a health care service.

## Patients and methods

### Registry-based cancer and non-cancer populations

**Fig 1:**
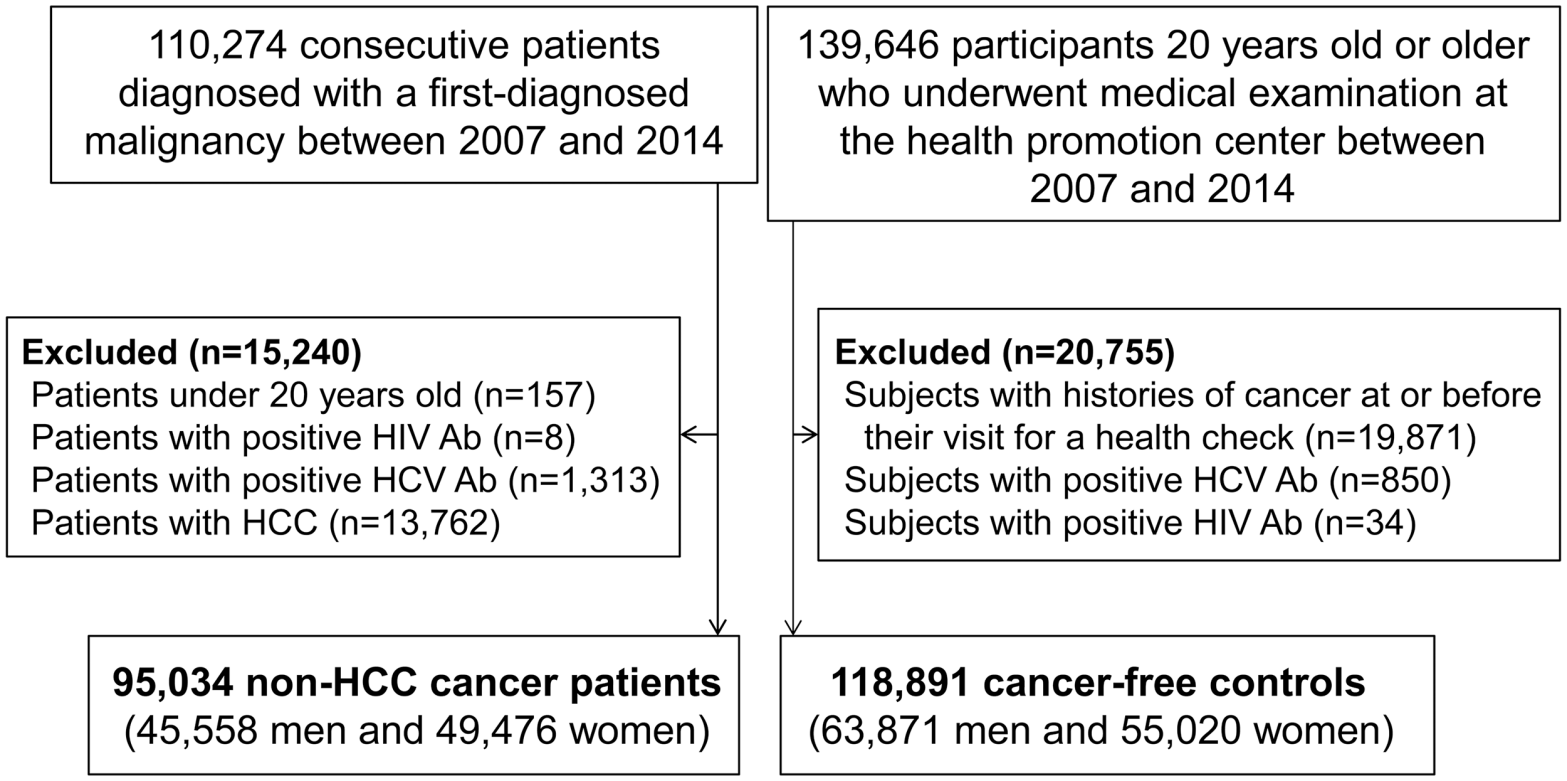
Flow diagram of the study.

In this retrospective registry-based case-control study, we identified 110,274 consecutive patients diagnosed with a first malignancy in a prospectively-constructed hospital-based cancer registry of Asan Medical Center between January 2007 and December 2014, as shown in Fig 1. This cohort registry was part of the National Cancer Registration Program initiated by the Ministry of Health and Welfare, Republic of Korea in 1980, and has continued to collect information on patient demographics including sex, age, date of diagnosis, cancer site, morphology, and pathological tumor type. Details of the cancer registry in our center, the largest tertiary referral hospital in Seoul, Korea, have been given elsewhere.[23] Of the consecutive patients, 15,240 were excluded for the following reasons: those under 20 years old (n = 157); those with human immunodeficiency virus antibody (n = 8) or hepatitis C antibody (n = 1,313); and those diagnosed with HCC, a well-established consequence of chronic hepatitis B (n = 13,762).[2] Finally, 95,034 new cancer patients (45,558 men and 49,476 women) were included in the main analyses of this study. All cases of malignancy were diagnosed based on histological confirmation of tissue or bone marrow. The diagnostic and staging work-ups for each patient were determined according to the standard protocol for the specific tumor type mainly based on guidelines recommended by the National Comprehensive Cancer Network (www.nccn.org).

To obtain controls as nearly as possible representative of the healthy population we used 139,646 subjects 20 years old or older who underwent medical examination at the health promotion center of our institution over the period of the study. Individuals with histories of cancer at or before their visit for a health check were excluded (n = 19,871) on the basis of assessment by self-questionnaire during the health checkup, hospital records, and data of the Korea National Cancer Registry and Korean National Health Insurance Service database, which have been validated as useful resources for research.[24] Subjects with human immunodeficiency virus antibody (n = 34) or hepatitis C antibody (n = 850) were also excluded. Altogether 118,891 individuals (63,871 men and 55,020 women) were selected.

The study was undertaken with the approval of the Institutional Review Board of Asan Medical Center (IRB No.2015-0513) and in accordance with the Declaration of Helsinki (1989) of the World Medical Association. The need for informed consent from the cancer patients was waived. According to the regulations of the National Health Insurance Scheme, which covers almost the entire population of Korea, it is mandatory for cancer patients to provide information to the cancer registry without specific agreement. In the case of the control participants, written informed consent for the use of anonymized personal data for research purposes was routinely obtained prior to the health screening. In this context, informed consent for this retrospective study was waived.

### Collection of cancer status data

The cancer registry data of our center contains demographic and clinical information including age, sex, time of diagnosis, cancer classification, and histologic type of cancer for all cases of cancer [23]. Using the International Classification of Diseases codes (version 10), we classified all malignant neoplasms as one or other of the following: head and neck (00–14, 30–32), esophagus (15), stomach (16), colon (18–19), rectum (20), gallbladder (23), intra- and extra-hepatic bile duct (22, 24), pancreas (25), lung (34), bone and soft tissue (40–41, 49), skin (43–44), breast (50), cervix (53), uterus (54–55), ovary (56), prostate (61), kidney (64–65), bladder (67), brain (71), thyroid (73), lymphoma (81–89), and hematologic malignancies including leukemia and multiple myeloma (90–96).

The following data for each patient were obtained from complete inpatient and outpatient medical records using the clinical database system of our institution (Asan BiomedicaL Research Environment, ABLE): presence of hypertension and diabetes, history of alcohol consumption and smoking, body mass index (BMI), and total cholesterol. The controls completed a health questionnaire at baseline that included information about daily alcohol and smoking consumption. Routine measurement of BMI and total cholesterol were carried out as part of the health check-up.

### Test for hepatitis B virus infection

Every cancer patient and individual attending for a health checkup in our hospital undergoes a blood test for HBV infection as part of the routine examination on the first visit. Because most HBV infections in Korea occur during the perinatal period or in early childhood, one measurement indicating hepatitis B surface antigen (HBsAg) positivity is regarded as reflecting chronic HBV infection.[25] Serum HBsAg was measured using the Architect assay (Abbott Laboratories, Chicago, IL, USA) or an immunoradiometric assay kit (DiaSorin, Vercelli, Italy).

### Statistical analysis

To examine the sero-epidemiological connections between infection with HBV and various types of cancer, we employed individual case-control matching with well-known confounders for cancer development.[26] That is, each patient in the case group was randomly matched with an individual in the control group provided they had the same profile in terms of smoking, hypertension, diabetes, and alcohol drinking, and only small differences in age (<3 year), BMI (<3 kg/m^2^) and cholesterol (<10 mg/dL). To analyze this matched data, we used the conditional logistic regression model. In addition, we conducted individual analyses of tumor subtypes for cancers that were found to be significantly associated with HBV infection. In the matched cohorts, the control group was regarded as the reference, and the odds ratios (ORs) together with 95% confidence intervals (CIs) are presented for each cancer subtypes. Univariate and multivariate multinomial logit models were fitted for significant cancer subtypes.

All statistical analyses were conducted using R software version 3.1.1 (R Foundation for Statistical Computing, Vienna, Austria, http://www.R-project.org/). In particular, the R packages for ‘survival’ [1] and ‘nnet’ [2] were used for the conditional logistic model and multinomial logit model fitting.[27, 28] All statistical inferences were two-sided, and a *P-*value less than 0.05 was considered statistically significant.

## Results

### Characteristics of the study and control populations

Table 1 shows the baseline characteristics of the cancer and control subjects. The median age of the cancer patients was 65 years (interquartile range [IQR] 56–73 years) for men and 56 years (IQR 47–66 years) for women. There were substantial differences between cases and controls in terms of age, smoking habits, alcohol consumption, BMI and total cholesterol level: men and women with neoplasms were more likely to be older, more hypertensive and diabetic than those without cancer; current smoking and alcohol use were more frequent in the control group than in the cancer patients; and the men with cancer had lower BMIs and lower levels of serum total cholesterol than the controls, while the women with cancer had higher BMIs. Women in both groups were less likely than men to be current or former smokers, and to consume any alcohol.

**Table 1:** Characteristics of the study populations.

**Pooled cohort**				
**Variable**	**Men**		**Women**	
	**Cases** **(n = 45,558)**	**Controls** **(n = 63,871)**	**Cases** **(n = 49,476)**	**Controls** **(n = 55,020)**
Age, years*	65 (56–73)	53 (46–60)	56 (47–66)	52 (44–59)
Hypertension	15,189 (33.3%)	12,155 (19.0%)	11,123 (22.3%)	6,447 (11.7%)
Diabetes	7,686 (16.9%)	5,199 (8.1%)	4,291 (8.7%)	2,092 (3.8%)
Any alcohol use	16,540 (36.3%)	40,791 (63.9%)	6,051 (12.2%)	10,510 (19.1%)
Smoking status				
Never	13,583 (29.8%)	15,002 (23.5%)	46,135 (93.2%)	50,599 (92.0%)
Former	22,070 (48.4%)	24,761 (38.8%)	2,171 (4.4%)	2,201 (4.0%)
Current	9,905 (21.7%)	24,108 (37.7%)	1,170 (2.4%)	2,220 (4.0%)
Body mass index, kg/m^2^*	23.8 (21.8–25.8)	24.3 (22.6–26.1)	23.2 (21.1–25.5)	22.0 (20.2–24.1)
Serum Cholesterol, mg/dL*	166 (139–193)	190 (168–214)	181 (156–207)	188 (166–212)
**Matched cohort**				
**Variable**	**Men**		**Women**	
	**Matched cases** **(n = 39,414)**	**Matched controls** **(n = 39,414)**	**Matched cases** **(n = 46,330)**	**Matched controls** **(n = 46,330)**
Age, years*	63 (55–71)	63 (55–70)	55 (46–64)	55 (46–64)
Hypertension	12,419 (31.5%)	12,419 (31.5%)	9595 (20.7%)	9595 (20.7%)
Diabetes	5779 (14.7%)	5779 (14.7%)	3293 (7.1%)	3293 (7.1%)
Any alcohol use	14,890 (37.8%)	14,890 (37.8%)	5555 (12.0%)	5555 (12.0%)
Smoking status				
Never	11,251 (28.5%)	11,251 (28.5%)	44333 (95.7%)	44333 (95.7%)
Former	19506 (49.5%)	19506 (49.5%)	1166 (2.5%)	1166 (2.5%)
Current	8657 (22.0%)	8657 (22.0%)	831 (1.8%)	831 (1.8%)
Body mass index, kg/m^2^*	23.9 (22.0–25.8)	24.0 (22.3–25.6)	23.1 (21.1–25.4)	22.8 (21.0–25.0)
Serum Cholesterol, mg/dL*	170 (147–195)	171 (148–195)	183 (159–208)	183 (160–208)

* Median (interquartile range)

Among the 45,558 men with cancer included in the study, stomach (n = 10,977), lung (n = 5,756), and colon (n = 4,101) cancers were the most common malignancies. Breast (n = 12,487), thyroid (n = 9,815) and stomach (n = 5,373) cancers were most frequent among the 49,476 women with malignancies. These results resemble the cancer incidence data in the national population-based cancer registry in Korea.[29]

In terms of matched set, baseline characteristics of the matched cohort, which included 85,744 subjects with malignancy and 85,744 healthy controls (1:1 ratio), are presented in Table 1. The distribution of common risk factors for the various cancers was comparable in the matched cohort.

### Association between hepatitis B virus infection and site-specific cancers

**Fig 2:**
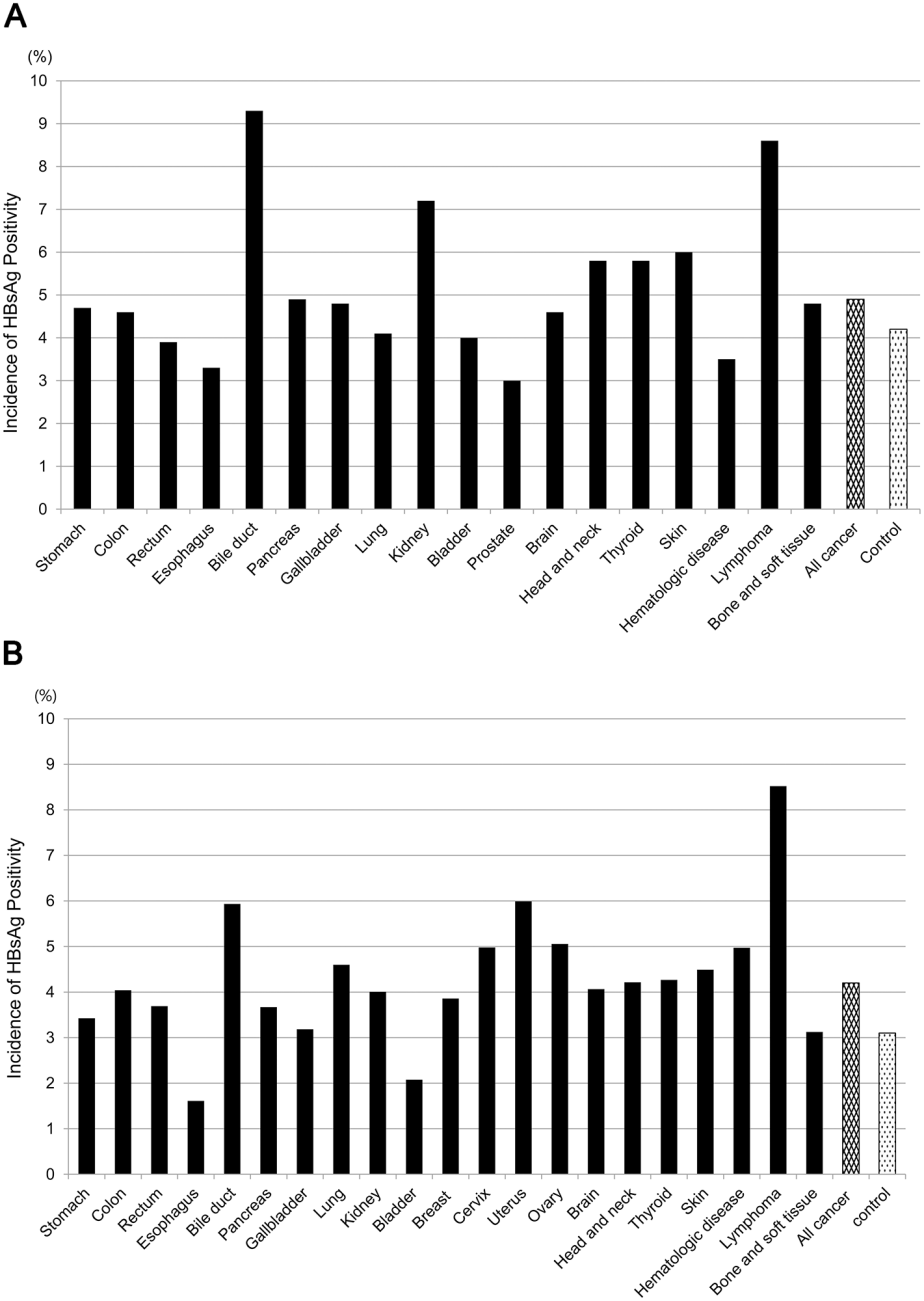
Prevalence of HBV infection by gender and primary tumor site among cancer patients and non-cancer subjects. HBsAg positivity was more frequent in cancer cases than in controls for both genders (4.9% *vs*. 4.2% for men; and 4.2% *vs*. 3.1% for women, both *P*s<0.001) (A) Male patients with bile duct cancer (9.3%), lymphoma (8.6%), and kidney cancer (7.2%) were more frequently infected with HBV than controls. (B) In women with malignances, positive HBsAg was more frequently seen in patients with lymphoma (8.5%), uterine cancer (6.0%), and biliary cancer (5.9%).

With regard to HBV-infected status, we identified 4,328 HBsAg-positive cancer patients (4.6%) of which 2,237 were men (4.9%) and 2,091 were women (4.2%). The sero-prevalence rate was significantly lower in the controls, with 4,401 HBsAg-positive subjects (3.7%) made up of 2,670 men (4.2%) and 1,731 women (3.1%) (*Ps*<0.001 for both gender). Fig 2A and 2B give the prevalence of HBsAg positivity in the cancer population according to specific tumor site. Positive HBsAg was relatively frequent in biliary cancer and lymphoma in both males (9.3% and 8.6%, respectively) and females (5.9%; and 8.5%, respectively), and the same was true for renal cancers in men (7.2%) and uterine cancers in women (6.0%).

In men, bile duct cancer (adjusted odds ratio [AOR] 2.59, 95% CI 1.98–3.39, *P*<0.001), lymphoma (AOR 1.53, 95% CI 1.12–2.09, *P* = 0.007), and dermatologic cancer (AOR 5.33, 95% CI 1.55–18.30, *P* = 0.008) were significantly correlated with HBV status, as shown in Table 2. In women, both biliary cancer (AOR 1.71, 95% CI 1.16–2.51, *P* = 0.007) and lymphoma (AOR 3.04, 95% CI 1.92–4.82, *P*<0.001) were significantly linked to HBV infection in the exact matching model with adjustment of cancer risk factors, which was consistent with the male data (Table 3). In terms of female-specific cancers, additional matching analyses yielded significant associations for breast (AOR 1.16, 95% CI 1.02–1.32, *P* = 0.033), cervical (AOR 1.49, 95% CI 1.11–2.00, *P* = 0.007), and uterine cancers (AOR 1.69, 95% CI 1.09–2.61, *P* = 0.019). HBsAg status was significantly correlated with lung (AOR 1.79, 95% CI 1.32–2.44, *P*<0.001) and thyroid cancers (AOR 1.49, 95% CI 1.28–1.74, *P*<0.001) in the matched women’s cohort, but not in the matched men’s cohort (AOR 0.91, 95% CI 0.75–1.11, *P* = 0.370 for lung cancer; and AOR 1.24, 95% CI 0.96–1.60, *P* = 0.104 for thyroid cancer).

**Table 2:** Relationship between hepatitis B virus infection and site-specific cancers in men.

Cancer Site	Number of patients in the pooled cohort		Matched analysis*		
	Total	HBsAg+	OR	95% CI	*P* value
Stomach	10,977	519	1.03	0.90–1.17	0.709
Colon	4,101	189	1.13	0.90–1.42	0.275
Rectum	3,293	127	0.82	0.63–1.05	0.120
Esophagus	1,089	36	1.00	0.62–1.62	1.000
Bile duct	2,622	245	2.59	1.98–3.39	<0.001
Pancreas	2,123	105	1.17	0.84–1.62	0.358
Gallbladder	545	26	0.94	0.48–1.86	0.862
Lung	5,756	236	0.91	0.75–1.11	0.370
Kidney	1,595	115	1.16	0.85–1.59	0.343
Bladder	1,273	51	0.96	0.63–1.45	0.833
Prostate	3,920	116	0.62	0.47–0.81	<0.001
Brain	1,586	73	0.90	0.65–1.26	0.553
Head and neck	1,323	77	1.33	0.94–1.88	0.113
Thyroid	2,510	146	1.24	0.96–1.60	0.104
Skin	282	17	5.33	1.55–18.30	0.008
Hematologic disease	1,009	35	0.46	0.29–0.74	0.002
Lymphoma	1,303	112	1.53	1.12–2.09	0.007
Bone and soft tissue	251	12	1.38	0.55–3.42	0.493

CI = confidence interval; HBsAg = hepatitis B virus surface antigen; OR = odds ratio.

* Controls were matched to cases by age, hypertension, diabetes, alcohol consumption, smoking status, and serum cholesterol level.

**Table 3:** Relationship between hepatitis B virus infection and site-specific cancers in women.

Cancer Site	Number of patients in the pooled cohort		Matched analysis*		
	Total	HBsAg+	OR	95% CI	*P* value
Stomach	5,373	184	0.98	0.79–1.21	0.825
Colon	2,921	118	1.15	0.87–1.52	0.320
Rectum	1,842	68	1.29	0.89–1.88	0.183
Bile duct	1,584	94	1.71	1.16–2.51	0.007
Pancreas	1,499	55	0.93	0.60–1.44	0.739
Esophagus	62	1	0.50	0.05–5.51	0.571
Gallbladder	691	22	1.06	0.55–2.05	0.866
Lung	2,653	122	1.79	1.32–2.44	<0.001
Kidney	674	27	1.16	0.63–2.14	0.640
Bladder	289	6	1.20	0.37–3.93	0.763
Breast	12,487	482	1.16	1.02–1.32	0.033
Cervix	2,370	118	1.49	1.11–2.00	0.007
Uterus	1,102	66	1.69	1.09–2.61	0.019
Ovary	1,048	53	0.67	0.34–1.31	0.240
Brain	2,484	101	1.09	0.82–1.47	0.549
Head and neck	427	18	1.33	0.63–2.82	0.451
Thyroid	9,815	419	1.49	1.28–1.74	<0.001
Skin	245	11	1.83	0.68–4.96	0.232
Hematologic disease	744	37	1.26	0.73–2.18	0.406
Lymphoma	974	83	3.04	1.92–4.82	<0.001
Bone and soft tissue	192	6	2.00	0.50–8.00	0.327

CI = confidence interval; HBsAg = hepatitis B virus surface antigen; OR = odds ratio.

*Controls were matched to cases by age, hypertension, diabetes, alcohol consumption, smoking status, and serum cholesterol level.

### Subtype analyses of HBV-related cancers

**Fig 3:**
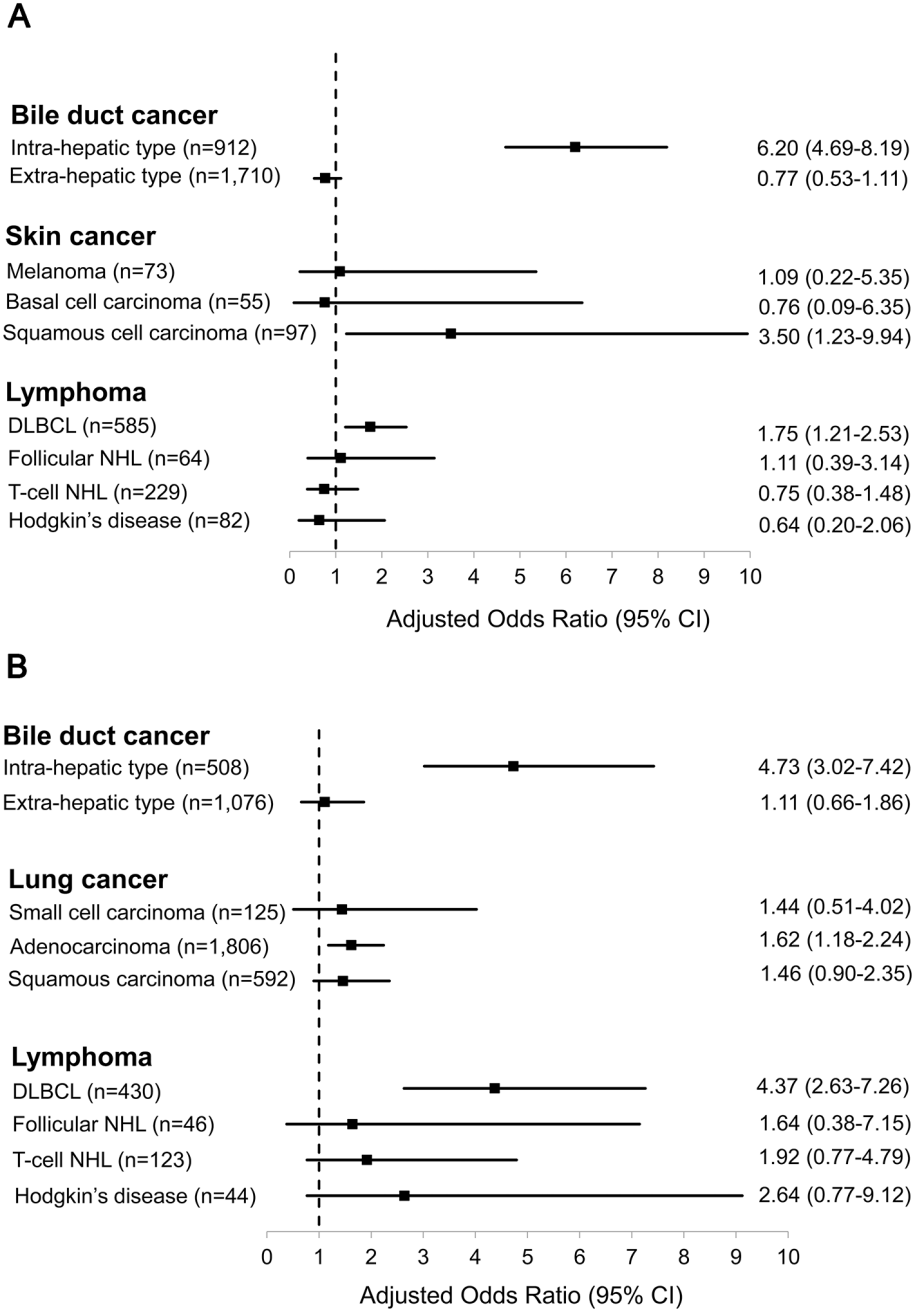
Subtype analyses of individual HBV-related cancers identified by the exact matching method in our series. (A) In men, the intra-hepatic type of bile duct cancer, DLBCL, and skin squamous cell carcinoma were significantly association with HBV infection (all Ps<0.05). (B) In women, lung adenocarcinoma as well as DLBCL and intra-hepatic cholangiocarcinoma were correlated with positive HBsAg (all Ps<0.05). No significant relationships were noted for other major sub-classification of the relevant cancers.

We next performed subgroup matched analyses for the individual cancer types identified as having significant associations with HBV infection by the conditional logistic regression method (Fig 3). For bile duct cancer, intra-hepatic cholangiocarcinoma (IHCCC), not extra-hepatic cholangiocarcinoma, was significant correlated with hepatitis B surface antigenemia in both genders (AOR 6.20, 95% CI 4.69–8.19, *P*<0.001 for men; and AOR 4.73, 95% CI 3.02–7.42, *P*<0.001 for women). In analyses based on the prevalent histological sub-types of the other HBV-related malignancies, there were significant positive links between HBV infection and diffuse large B cell lymphoma (DLBCL) (AOR 1.75, 95% CI 1.21–2.53, *P* = 0.003 for men; and AOR 4.37, 95% CI 2.63–7.26, *P*<0.001 for women); squamous cell carcinoma for skin malignancies in men (AOR 3.50, 95% CI 1.23–9.94, *P* = 0.019); and adenocarcinoma for lung cancer in women (AOR 1.62, 95% CI 1.18–2.24, *P* = 0.003). No significant correlation was observed for the remaining subtypes of the relevant cancers.

## Discussion

Although HBV is mainly thought to be hepatotrophic and hepatocarcinogenic,[30] the current analysis of matched data for a broad spectrum of cancers in a prospective cancer registry demonstrates that chronically-infected HBV status is closely associated with a variety of non-HCC malignancies such as lymphoma and biliary cancer, regardless of patient’s gender; cervical, uterine, breast, thyroid and lung cancers in females; and skin cancers in males.

Although one case-control study conducted in the U.S. with low HBV prevalence showed no associations,[31] several previous studies have suggested that HBV may play a causal role in the development of lymphoma, especially DLBCL, which is the most common and aggressive type of NHL.[5, 32] Our analysis also revealed that, of the various lymphoma subclasses, DLBCL had the strongest linkage with positive HBsAg (AOR 1.75 for men and 4.37 for women). Although little is known of how HBV may generate tumors, possible routes may be inferred from the known mechanisms by which HCV produces lymphomas, such as chronic B-cell proliferation in response to antigenic stimulation, high-affinity interaction between viral envelope and B-cell receptors and direct viral infection of B cells.[18, 33]

In our series, hepatitis B surface antigenemia increased the risk of IHCCC ---but not of extra-hepatic biliary neoplasms ----by 6.2- and 4.7- fold for men and women, respectively. Similar findings have often been reported in other population-based and case-control studies in different regions.[6, 32, 34] Active HBV replication and viral integration into the human genome within IHCCC tumors may provide the basis of the effect, since molecular genetic studies have demonstrated the presence of HBV DNA in IHCCC tissue specimens.[35, 36] Recent experiments also suggest that because progenitor cells can differentiate into human hepatocytes and cholangiocytes, HBV may induce cholangiocytic carcinogenesis by the same mechanism as it does for hepatocyte carcinogenesis.[34, 37] In addition, bile duct damage and fibrosis mediated by HBV-inducing chronic liver disease may be a potential process leading to IHCCC.[34, 35]

In contrast with our observations in males and females, several cancerous conditions, including pancreatic and gastric cancers that are of widespread health interest, have been shown to be significantly associated with HBV positivity.[4, 16, 20, 38, 39] A study by Hassan and colleagues from an HBV non-endemic country reported a relationship of pancreatic cancer with prior hepatitis B infection and positive anti-hepatitis B virus core (HBc) antibody, rather than current infection and positive HBsAg.[4] Also, Taiwanese and Swedish population cohort studies and Chinese case-control studies found only marginal associations between pancreatic cancer and the presence of HBsAg.[16, 20, 39] On the other hand, in contrast to our cohort from Korea where gastric cancer ranked 2nd in incidence, Chinese samples with stomach cancer were more frequently related to current HBV infection, compared to individuals with benign gastric diseases or other hospitalized subjects.[7, 16] These discrepant features of the association between hepatitis B viremia and foregut malignancies may be due to differences in study design, populations investigated, the incidences of cancer or hepatitis, and periods when the observations were made.

Our investigation appears to be the first to find evidence that chronic HBV infection is correlated with the presence of gynecologic, endocrinologic, pulmonary, and dermatologic cancers, which were little studied in prior investigations of this kind. Although the underlying mechanisms have not been clearly identified, some biological phenomena may be relevant: 1) integration of both HBV and human papilloma virus (HPV) into the same sites in the human genome, as well as inappropriate responses of human leukocyte antigen and lymphocytes to the viruses;[40, 41] 2) association of prolonged viral hepatitis with chronic inflammatory impairment of other organs leading to chronic obstructive pulmonary disease, autoimmune thyroiditis, and lichen planus of the skin with malignant potential;[42–45] and 3) detection of HBV DNA and HBV surface antigens in skin tissue and breast milk.[10, 46, 47] In relation to HBV-associated lung cancer, the bloodstream may be a possible route for HBV transmission, given the blood-borne transmission of HPV suggested by the development of adenocarcinoma of the lung.[47] Although correlations were revealed in the present study, further basic and clinical investigations will be needed to establish whether chronic HBV infection plays a substantial oncogenic role on the development of the various malignancies.

Our findings suggest important clinical consequences. The key point raised by this study is the need for a risk-adapted strategy for serologic testing of hepatitis B in patients with potentially associated cancers in order to prevent accidental HBV reactivation and hopefully to delay tumor progression, although this effect has not yet been proven other than in HBV-related HCC. However, the current U.S. Preventive Services Task Force recommendations do not advocate screening for hepatitis B in any cancer patients.[48] Conversely, it may be desirable that possible target cancers, no doubt not restricted to HCC, be regularly included in surveillance programs of individuals with chronic hepatitis B according to their individual associations with HBV. The cost-effectiveness of the proposed policies must be established prior to actual clinical application.

Some limitations of our cross-sectional study should be considered. Time-dependent longitudinal observation studies, together with experimental evidence, are needed to clarify the causal relationship between viral infection and the subsequent development of specific cancers. Another consideration is the relationship between resolved HBV infection without presence of the surface antigen and malignant conditions, which was not examined in the present study. Indeed, over 65% of the Korean general population are sero-positive for anti-HBc,[25] and hence anti-HBc status is not routinely checked in general clinical practice. In addition, we did not fully take into account all specific cancer-related causal factors, such as *Helicobacter pylori* for gastric cancer and HPV for cervical cancer.[26] Future studies should be investigated to investigate the additional effect of hepatitis B virus on the specific pathogen-induced carcinogenesis. However, in our analyses we adjusted common risk factors of the various cancers as confounders (i.e., age, BMI, alcohol consumption, cigarette smoking, diabetes, hypertension, and serum cholesterol), more comprehensively than other relevant studies.[4, 5, 7, 16, 19–22] Lastly, since control subjects had voluntarily visited the hospital for a general health check-up, there is a potential for selection bias. The matched analysis using conditional logistic regression used in this study may reduce the possibility of the bias.

In conclusion, chronic and current infection with HBV may be positively associated with the presence of several non-hepatocellular malignancies, including cervical and uterine cancers and even breast, thyroid, lung and skin cancers that have not previously been studied in this context. These findings should provide helpful practical information relevant to both HBV screening in cancer patients and cancer surveillance in hepatitis B patients.

## Supporting information

S1 File Raw data of this study. This file contains raw data about clinical variables of each patient in this study.
(XLSX)
